# The Cost of Caries in Primary Dentition: A Case Report of a Rare Cause

**DOI:** 10.1002/ccr3.70072

**Published:** 2025-01-06

**Authors:** Vladimir Mitic, Sasa Cvetkovic

**Affiliations:** ^1^ Department of Orthopedics of Jaws and Teeth, Faculty of Medicine University of Nis Nis Serbia; ^2^ Department of Oral Surgery Clinic for Dental Medicine Nis Serbia

**Keywords:** impactions, maxillary canines, orthodontic anchorage, orthodontic‐surgical therapy, primary molars, proximal caries

## Abstract

A 10.2‐year‐old girl, accompanied by her parents, came to the Department of Jaw Orthopedics at the Clinic for Dental Medicine. Based on the initial orthopantomography, multiple tooth impactions were diagnosed. In the upper jaw, the impaction of the canine and the second premolar on the left side was observed, along with.

**Summary**

- The presented case indicates much more difficult and complex conditions that need to be solved if distal proximal caries of the maxillary second primary molars occurs before the eruption of the first permanent molars.

## 1 Introduction

The eruption of permanent teeth is usually accelerated by the resorption of the jawbone, root of the milk tooth, and alveolar mucosa [1, 2]. Premature loss of primary teeth is one of the factors in the emergence of orthodontic irregularities, especially primary second molars before the first permanent molar erupts. Untreated caries, especially of the proximal surfaces of primary second molars, before the eruption of the first permanent molars can lead to mesial displacement of permanent teeth into the empty space during eruption [3, 4, 5] In recent years, there has been a change in the approach to the restoration of caries of milk teeth, with an emphasis on a biological approach compared to conventional procedures [6].

Biological approaches indicate the preservation of the tooth structure and maintaining the functionality of the dentoalveolar structures for as long as possible, while in the case of primary teeth, until the moment when their replacement with permanent replacement teeth is necessary. Such approaches fall under the domain of Minimal Intervention Dentistry (MID) [7]. When and in what way to use certain methods of treatment of carious lesions of primary teeth should be based on modern understandings of restorative dentistry with an adequate approach to young children. Sometimes, it is necessary to have a consensus of experts in order to choose the most favorable and acceptable way of treatment of caries in patients, including minimally invasive dental intervention [8].

A variety of factors can lead to the failure of teeth to erupt into the oral cavity. Mechanical obstructions such as the presence of other teeth, persistence of primary teeth, calcification of the surrounding bone, bad habits such as thumb or finger sucking, as well as conditions in which cement fuses with the adjacent bone can lead to ankylosis, preventing further eruption and tooth eruption [9].

Ankylosis of primary teeth with the presence of approximal caries can lead to the shortening of the dental arch, with the possibility of creating conditions for the impaction of replacement teeth [10]. The most frequently impacted teeth are the maxillary canines, where the etiological factors for this type of irregularity are numerous and varied. Early diagnosis of impacted teeth is of great importance in order to minimize costs, treatment time, and avoid possible complications [11, 12].

The presented case shows the untimely repair of occluso‐distal caries of the primary second molar and the consequences that arose, as well as the plan and the entire therapy of liberation of impacted teeth in the patient over a five‐year period.

## 2 Case History

**FIGURE 1:**
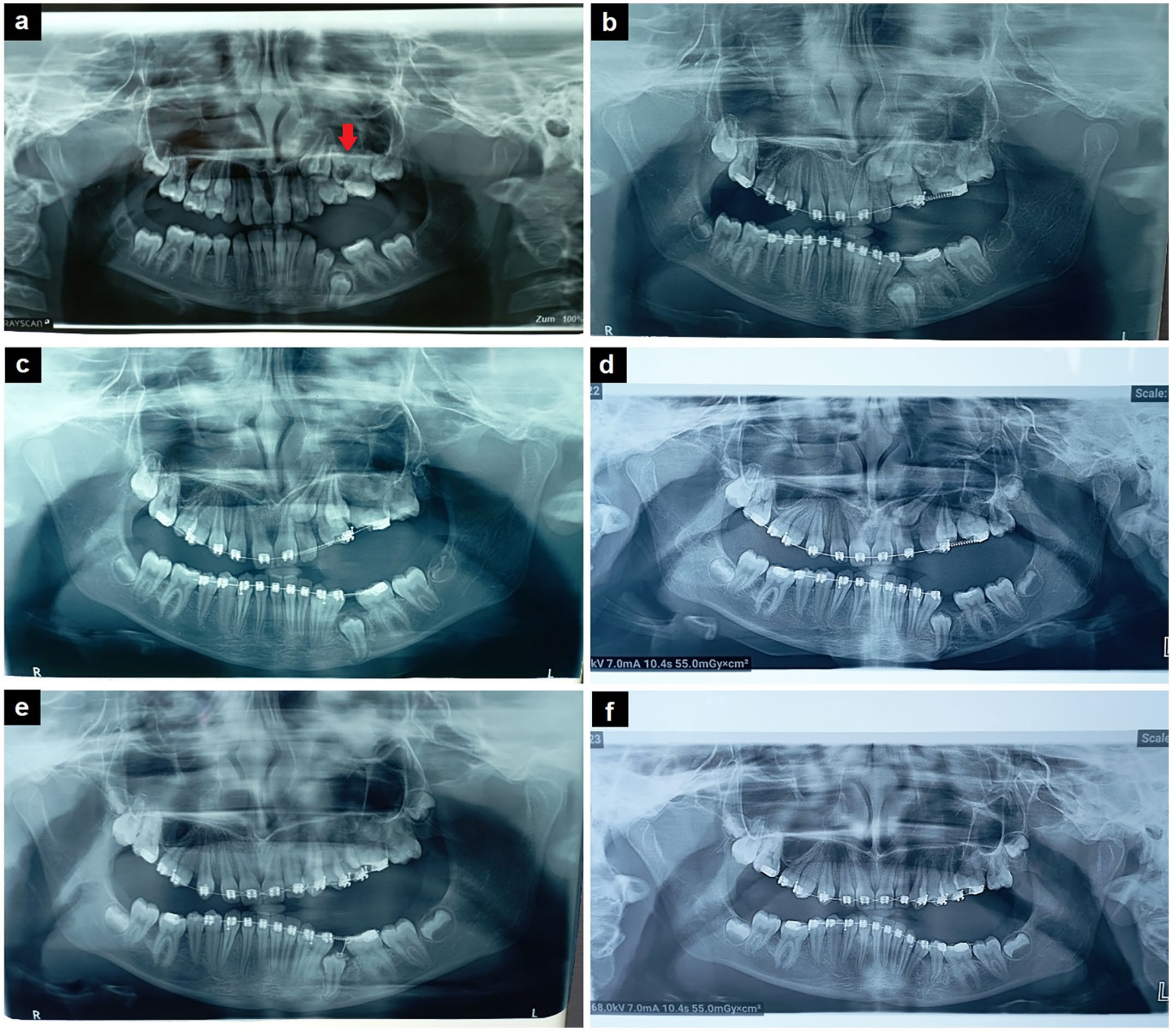
A 4‐year follow‐up with panoramic images: (a) Initial panoramic image (the age of the patient—9.10 years‐ impaction of primary second molar‐ the red arrow); (b) Panoramic image after orthodontic fixed appliances were placed (10.6 years); (c) Panoramic image after the extraction of primary second molar and the beginning of liberation of the impacted second premolar on the left side in the upper jaw (11.1 years); (d) Panoramic image after the second premolar was placed in the maxillary dental arch (11.5 years); (e) Panoramic image after all teeth were placed in the upper dental arch and the beginning of the second premolar liberation in mandibular arch (12.5 years); (f) Panoramic image after all teeth were placed in dental arches (13.11 years).

Accompanied by her parents, a 10.2‐year‐old girl came to the Department of Jaw Orthopedics at the Clinic for Dental Medicine in Nis, and on the orthopantomography image they brought with them (Figure 1a), multiple impacted teeth were seen. The impaction of the maxillary canine and the second premolar on the left side was observed with the simultaneous impaction of the primary second molar with visible destruction of the occluso‐distal surface of the crown of this tooth. It was concluded that caries of the primary tooth with ankylosis occurred before the eruption of the permanent first molar. During the eruption of the first permanent molar in the space created by the destruction of the distal surface of the primary second molar by caries, retention and impaction occurred. The contact of the first premolar and the first permanent molar on the left side in the upper jaw was present. A lateral open bite on the left side was also noticeable. In the lower jaw, an impaction of the second premolar was diagnosed with a mesial inclination of the first permanent molar on the left side. Written informed consent was obtained from the parents upon detailed explanation of the treatment procedure. Written informed consent was obtained from the patient and her parents to publish this report in accordance with the journal's patient consent policy.

## 3 Diagnosis and Treatment

Therapy was started by placing metal orthodontic brackets (Mini Sprint 0.022″, Forestadent, Germany) and tubes on all teeth except the second molars and the lateral incisor in the upper jaw on the left side (Figure 1b) and the initial arch wires 0.014″ (Titanol‐Budget, Forestadent, Bernhard Foster GmbH, Pforzheim, Germany). After bonding, a Ni–Ti open coil (American Orthodontic, Sheboygan, Wisconsin, USA) was placed in the upper jaw between the left central incisor and the first premolar, of the same length as the arch distance. An open spring was placed between the first premolar on the same side and the first permanent molar, 2 mm longer than the distance between these two teeth. Regular controls were performed every 2 weeks, during which only the open spring between the first premolar and the first permanent molar was changed and a new one was placed, always longer than the previous one by 2 mm. Thirteen months after bonding, surgical extraction of the impacted maxillary second primary molar from the palatal side was performed. After removing the sutures, the distalization of the upper left first permanent molar continued with the leveling of the dental arches and straightening of individual teeth, after which the surgical‐orthodontic liberation of the impacted upper second premolar was performed. After lifting the flap from the buccal side, the removal of the bone above the labially placed crown of the impacted second premolar was started (Figure 1c). After removing the excess bone from the crown and performing hemostasis and toileting, an orthodontic button (World Class Technology Corporation, USA) with a sterile twisted wire (Forestanit Super weich, Forestadent, Germany), with a diameter of 0.5 mm, was placed on the exposed surface of the impacted tooth, which was previously acid‐etched using 37% orthophosphoric acid gel for 20 s (Etching Gel, 3M, Monrovia, CA, USA), after which the tooth enamel surface was washed and dried. A primer (Transbond XT Primer, 3M Unitek, Monrovia, CA, USA) was applied over the button base with a microbrush (Disposable Micro Applicators, Med Comfort, Ampri GmbH, Germany), then adhesive (Transbond XT Paste, 3M Unitek, Monrovia, CA, USA) was applied over the primer and base, and finally the primer was applied over the adhesive on the button base. Immediately before placing the button on the tooth, an LED light (Mach LED 130, Edersberg, Germany) was moved to the side on the operating unit in the operating room to avoid premature polymerization. Polymerization was performed with a Woodpecker Dental Curing Light (LED B. Curing Light, Guangxi, China) for 20 s. Orthodontic‐surgical liberation of the maxillary canine was performed using the same technique after 8 months from the first intervention (Figure 1d).

In the lower jaw, in order to obtain an adequate result with a smaller number of surgical interventions, a wait‐and‐watch approach was applied, in which the emergence of the lower second premolar on the left side was expected after the distalization of the first permanent molar and a created space. Since such a scenario did not occur due to the improper development of the apical part of the root of the second premolar (Figure 1e), orthodontic‐surgical liberation of this tooth was performed.

Surgical liberation of the lower second left premolar was performed with an open flap and placement of a twisted wire with a loop on the buccal surface of the tooth by applying a primer (D‐Line, Light Curing Adhesive, Medicinos Linija UAB, Lithuania) in a thin layer with a brush (3M ESPE, Disposable Applicator, Brush Tips, St. Paul, USA) and its polymerization. A liquid composite was applied (D‐Line, Light Curing, Nano, Flowable, Composite, Medicinos Linija UAB, Lithuania). Immediately before the polymerization of the composite, the wire with the loop was immersed in the composite, and polymerization was carried out. The free twisted portion of the wire was wrapped around a 0.016 arch (G & H Wire Co., Indiana, USA) in the lower arch of teeth.

The panoramic image after completed orthodontic‐surgical interventions of liberation of the impacted teeth shows an adequate intradental but not interdental relationship (Figure 1e,f). Also, the condyles on the initial panoramic image were not at the same levels, while at the end of therapy and the liberation of all three impacted teeth, their consolidation and position in the fossae, as well as their mutual position, which became symmetrical, could be observed. The complication in the form of a lateral open bite was an inevitability that occurred due to the reduced ability of the patient to eat on that side.

In order to avoid additional pressure on the first premolar on the left side in the upper jaw, a reduced orthodontic palatal appliance was made with lateral filarets on the left side along with two wire pins, which would redirect the traction towards the palate with interdental elastics.

## 4 Discussion

In orthodontics, the loss of primary second molars before the eruption of the first permanent molars represents an enigma and a challenge in the therapy of orthodontic irregularities. Preserving the proximal surfaces of the crown of primary molars is of vital importance, especially on the distal side, during the eruption of the first permanent molar, which Mazhari [13] described and illustrated. An actively erupting permanent first molar will migrate mesially if a primary deciduous molar is missing, causing a localized loss of space, along with a significant discrepancy between space and tooth size. At the same time, on the initial orthopantomography of the patient, differences in premature tooth loss can be recognized before the eruption of the first permanent molar (upper jaw) and after the eruption of the first permanent molar (lower jaw). The consequences that occur are far more complex before the eruption of permanent molars, where only the distal surface of the primary second molar was lost and not the whole tooth. Such cases call for caution as well as timely detection of caries in the primary dentition and their rehabilitation, where not only the health condition of primary teeth is affected, but adequate conditions are created for the eruption of permanent teeth. This also prevents possible complications that may arise in primary dentition and, as such, will be manifested in the form of consequences threatening permanent dentition that will require numerous radical surgical interventions with complex and long‐term therapy with more expensive and sometimes bulky appliances.

Options for therapy were:

- Surgical extraction of the impacted primary second molar with simultaneous extraction of the first premolar in the upper jaw on the left, with subsequent liberation of the impacted canine and second premolar;
- Surgical extraction of the primary second molar with the extraction of the first permanent molar with subsequent liberation of the impacted teeth;
- The use of temporary anchorage devices (TADs) was initially rejected due to the necessary movement of the teeth during treatment.

The first two options were initially excluded due to the increased intradental space that would have been created by tooth extractions due to the later applied traction on the arches during the liberation of impacted teeth. The third option, which was reluctantly accepted by the patient and parents as another invasive intervention, was rejected due to the necessary tooth movements, primarily the distalization of the first permanent molar and the still unerupted second permanent molars. The decision about therapy was made in agreement with the parents to try to preserve all permanent teeth.

In our case, we used the first premolar in the form of a “natural implant” as an anchorage to bring both teeth into the upper dental arch. This tooth, with the help of an open coil spring placed up to the central incisor, first suffered horizontal forces distalizing the first permanent molar and then vertical forces when pulling the second premolar. After a break of 3 months because a slight luxation of the first premolar was noted, pulling of the canine with mild vertical forces continued until its final placement in the dental arch. Special attention was paid to the preparation of the button with a twisted wire. A space was left at the point of contact so that the wire did not separate from the button when pulling, while at the same time it was possible to direct the wire in the required direction [14] in the operating room by an orthodontist. In this way, the possibility of eventual disconnection of the button from the tooth was reduced, thus minimizing the possibility of reoperation.

The modified method of direct orthodontic bonding [15] (brackets, tubes, and buttons) was chosen because it allowed constant visual inspection into possible contamination of demineralized tooth surfaces.

The total costs of orthodontic‐surgical interventions up to the moment of placing all the teeth in dental arches was 1147.70 $, which represents the internal amount (without the external costs of traveling to regular check‐ups) that was necessary when providing health services at our clinic. The restoration of caries of the primary tooth at the time of the patient's presentation amounted to 2.1 $, with a note that a certain amount of money (17.9% or 125 $) was covered by the Republic Institute for Health Insurance of the Republic of Serbia, while the remaining amount (89.1% or 1022.7 $) was set aside by the family to cover the costs of non‐standard dental interventions.

Given that the therapy lasted 5 years with four surgical interventions, the necessity of a good diagnosis, a precise therapy plan, and harmonized attitudes between doctors, but also understanding and consent of parents and patients, is crucial in obtaining a satisfactory result. Therefore, the applied therapy of the presented patient should not be part of a routine work protocol. Instead, a complete treatment plan should be based on a carefully selected type of device and intervention that should be comprehensively designed and carefully applied.

## 5 Conclusion

- Timely diagnosis and rehabilitation of primary teeth must be imperative for the entire dental medicine system.
- A preventive approach, along with additional education of parents and children, is very important in order to avoid numerous complications and radical multi‐disciplinary treatments over a longer period of time.
- The clinical and economic costs are certainly important; however, the time lost in solving the later consequences, especially those minor conditions such as caries of primary teeth, which initially require minimal or non‐invasive therapy, is even more important.

## Author Contributions

**Vladimir Mitic:** conceptualization, investigation, methodology, supervision, validation, visualization, writing – original draft, writing – review and editing. **Sasa Cvetkovic:** investigation, methodology, supervision, validation.

## Data Availability

The data that support the findings of this study are available from the corresponding author upon reasonable request.
